# Association of the Inactive Circulating Matrix Gla Protein with Vitamin K Intake, Calcification, Mortality, and Cardiovascular Disease: A Review

**DOI:** 10.3390/ijms20030628

**Published:** 2019-02-01

**Authors:** Stefanos Roumeliotis, Evangelia Dounousi, Theodoros Eleftheriadis, Vassilios Liakopoulos

**Affiliations:** 1 Division of Nephrology and Hypertension, 1st Department of Internal Medicine, AHEPA Hospital, School of Medicine, Aristotle University of Thessaloniki, 54636 Thessaloniki, Greece; st_roumeliotis@hotmail.com (S.R.); teleftheriadis@yahoo.com (T.E.); 2 Department of Nephrology, Medical School, University of Ioannina, 45110 Ioannina, Greece; evangeldou@gmail.com

**Keywords:** calcification, cardiovascular disease, dpucMGP, matrix Gla protein, mortality, renal function, vitamin K.

## Abstract

Matrix Gla Protein (MGP), a small Gla vitamin K-dependent protein, is the most powerful natural occurring inhibitor of calcification in the human body. To become biologically active, MGP must undergo vitamin K-dependent carboxylation and phosphorylation. Vitamin K deficiency leads to the inactive uncarboxylated, dephosphorylated form of MGP (dpucMGP). We aimed to review the existing data on the association between circulating dpucMGP and vascular calcification, renal function, mortality, and cardiovascular disease in distinct populations. Moreover, the association between vitamin K supplementation and serum levels of dpucMGP was also reviewed.

## 1. Introduction

Vascular calcification (VC) is an ongoing process that starts in childhood and develops as humans grow. It may occur in advanced age as a natural phenomenon, but in conditions with high atherogenic status—such as diabetes [1], oxidative stress [2,3], and chronic kidney disease (CKD) [4]—it may appear earlier, in a greater degree, and is associated with increased cardiovascular (CV) mortality and morbidity. For a long period of time, VC was regarded as a passive, degenerative accumulation process of calcium and phosphate in the arterial wall without any treatment options. This theory was first suggested by Virchow et al. in 1858 [5], and it prevailed for over a century. Recently, our perspective changed, when accumulating evidence pointed towards an active, complex condition where differentiation of vascular smooth muscle cells (VSMCs) to an osteoblastic phenotype is the first, crucial step initiating the calcification process. Moreover, it was suggested that Gla containing proteins that are involved in bone metabolism play an important role in regulating VC [6]. In recent years, research has been focused on the recognition of the function and regulation of novel proteins accelerating or inhibiting VC. 

## 2. Types of Vascular Calcification

Calcification occurs in small and large arteries (carotid, aorta, femoral), soft tissues, and heart valves. VC found in any human artery increases the risk for CV disease and death by 3.4 [7]. It is well-established that human biological age is directly related to the conditions of the vasculature [8]. Morphologically, two types of VC have been observed: atherosclerotic intimal calcification that develops near cholesterol deposits in atheromatic plaques and leads to atheromatosis, and arterial medial calcification, which is characterized by calcific deposits within the media artery layer [9]. Intimal calcification starts in the inner layer of large arteries (mainly aorta and coronary arteries), progressively expands in the medial tissue, is associated with dyslipidemia, and causes ischemia and arterial infarction. Calcification of the medial layer occurs even in small arteries irrespective of intimal atheromatosis, is associated with gradual VSMC differentiation to osteoblastic phenotype, and leads to arterial stiffness, hypertension, and left ventricular hypertrophy [10]. Cardiovascular calcification can be found in the tunica intima and medial aortic tissue, the aortic arch or branches, the coronary arteries, the myocardium, and the heart valves.

## 3. Matrix Gla Protein

### 3.1. Role and Activation

Matrix Gla Protein (MGP) is a small 12 kDa, 84 amino acid Gla protein that belongs to the vitamin K-dependent proteins (VKDPs) and is secreted by chondrocytes and VSMCs. It was first discovered by Price et al. in bones, but is also expressed in the heart, vessels, kidneys, and cartilage [11,12,13]. MGP is the first protein recognized as an inhibitor of VC both in vitro and in vivo, and is considered the most powerful natural inhibitor of calcification in the human body [14,15,16]. Moreover, this small protein is the only known factor that not only inhibits, but can also reverse the calcification process.

MGP protects from artery calcification through several pathophysiological mechanisms. Firstly, it has a high binding affinity to newly formatted hydroxyapatite crystals, and therefore abrogates their accumulation within the arterial wall. Secondly, after binding with calcium and phosphate crystals, it upregulates the arterial macrophages to promote phagocytosis and apoptosis of the MGP-hydroxyapatite complex [17]. Thirdly, MGP directly inhibits binding of bone morphogenetic protein-2 (BMP-2) to its receptor, and subsequently downregulates its function [18,19]. BMP-2 is expressed in endothelial foam cells of human atherosclerotic plaques [20], leading to chondrogenesis, osteogenesis, and VC by inducing the osteoblast differentiation of VSMCs within the arterial wall. BMP-2 can promote osteoblast transformation only after binding to its high affinity receptor [21,22]. Results from animal models showed that the MGP/BMP-2 complex exists in vivo, suggesting that MGP inhibits the functions of BMP-2. In calcified arteries of aging rats, MGP was undercarboxylated and could not bind BMP-2 [23]. MGP affinity to hydroxyapatite is directly dependent on the concentration of free calcium ions in a dose-dependent manner: 1 mmol/L increase in calcium levels results in a two-fold increase in MGP-hydroxyapatite binding affinity [24]. MGP is a VKDP with Gla and serine residues. To become biologically active, MGP has to undergo γ-carboxylation of the Gla residues [25,26,27], followed by phosphorylation of the serine residues [28]. Carboxylation and phosphorylation of MGP depend on vitamin K as their substrate [25,26,27]. Only after carboxylation, MGP undergoes a biochemical change in its structure, crucial for its binding to BMP-2 and calcium crystals [27]. Vitamin K deficiency leads to inactivation of MGP and acceleration of VC, while high vitamin K intake can reverse both conditions [29].

### 3.2. MGP Forms

Low levels of vitamin K2 (especially menaquinone-7, MK-7) result in various inactive forms of MGP, according to its state of carboxylation and/or phosphorylation: the uncarboxylated MGP (ucMGP), carboxylated but not phosphorylated MGP (dpcMGP), phosphorylated but uncarboxylated (pucMGP), and the fully inactive uncarboxylated, dephosphorylated MGP (dpucMGP), where the active form is both phosphorylated and carboxylated. For this narrative review, we searched the Medline database with the key words “Matrix Gla protein,” “MGP polymorphism,” “inactive MGP,” “dephosphorylated MGP,” “uncarboxylated MGP,” and “vitamin K and MGP,” and we selected all trials that studied possible association of MGP forms and polymorphisms with vascular calcification, adverse events, and vitamin K supplementation.

### 3.3. MGP and VC

The first study suggesting that MGP is the most powerful VC inhibitor in the human body was conducted in 1997. Luo et al. developed a knockout mouse model with genetic deficiency of MGP (MGP −/−) and found that, although born with normal phenotype, after 6–8 weeks these mice developed severe VC and died of aortic plaque rapture and subsequent internal massive hemorrhage [14]. The authors suggested that MGP deficiency is not compatible with life. MGP deficiency has been repeatedly associated with significant changes in structure and function of extracellular matrix Gla and subsequent accelerated VC in several knockout MGP animal models [30,31]. Several studies have suggested that MGP is overexpressed in the walls of atherosclerotic arteries [32,33,34,35] and in humans, calcified atheromatic plaques [32]. Moreover, VC severity—assessed by electron beam computer tomography—was directly associated with MGP serum levels. The fact that mutation in the gene encoding MGP causes Keutel syndrome, a rare autosomal recessive disorder characterized by severe soft tissue calcification, also suggests a pivotal role of MGP in VC prevention [15].

### 3.4. Uncarboxylated Form of MGP: An Early VC Biomarker

Schurgers et al. developed specific antibodies against uncarboxylated MGP and showed that ucMGP is accumulated in calcified vessels of diabetics [18]. Roijers et al. used a novel, highly specific proton microscope to detect microcalcification in human vessels—that is, a very small amount of calcium and phosphorus that could not be measured until now by electron microscope. Based on the severity of microcalcification, the authors histologically divided human coronary arteries into 5 categories, 1 being normal artery and 5 those with the most severe calcification. UcMGP concentration gradually increased along with VC categories [36]. Moreover, even in category 1 arteries—considered healthy—there was already a significant amount of microcalcification. Similarly, ucMGP was strongly correlated with VSMCs differential and calcium accumulation in the wall of human coronary arteries [37].

There is a growing body of in vivo evidence suggesting that ucMGP is associated with VC. After 6 weeks of treatment with warfarin, rats developed severe artery calcification [38]. For the next 6 weeks, the animals were allocated to receive warfarin and vitamin K (group 1), low vitamin K (group 2), or high vitamin K (group 3). At the end of the study, arterial calcium levels were even more increased in the warfarin group and slightly increased in the second group. On the contrary, the high Vitamin K group had a 37% improvement in arterial elasticity. Moreover, ucMGP levels were significantly higher in the warfarin group compared to the two other groups. Rats treated with high vitamin K had the lowest concertation of ucMGP. The authors of the study suggested that ucMGP reflects vitamin K deficiency and is a novel marker for VC, but also that vitamin K intake can reverse the calcification process [38]. Similarly, ucMGP was detected in calcified aortas of uremic rats [39]. Compared to rats with normal renal function, ucMGP was overexpressed in noncalcified (5-fold) and calcified (20-fold) aortas of uremic rats [39].

In human studies, serum ucMGP levels of 172 patients with severe coronary artery disease (CAD) were significantly lower compared to healthy normal controls [18]. Likewise, plasma ucMGP concentration was strongly correlated with calcium deposits in abdominal aorta, common carotid, and coronary arteries of 36 hypertensive subjects [40]. In 571 healthy women, serum ucMGP levels were not correlated with the presence of coronary artery calcification (CAC) [41]. Similarly, in a cohort of 438 healthy elders, there was no association between serum ucMGP and carotid intima-media thickness (cIMT), pulse wave velocity (PWV), or CAC score. Data from the Heart and Soul study showed that in patients with stable CAD, low plasma ucMGP concentrations were independently associated with mortality and CV events [42]. 

On the other hand, carboxylated MGP exerted a protective function in pathogenesis of calciphylaxis. In patients undergoing maintenance hemodialysis (HD), a decrease of 0.1 in serum cMGP was associated with a two-fold risk of developing calciphylaxis [43]. In HD patients, several investigators showed that ucMGP plasma levels are significantly higher compared to general population and are strongly inversely linked with VC [44,45,46]. However, in HD children, ucMGP was not associated with VC [47].

Since MGP is expressed in the kidney 5-fold more than in bones [48], it was hypothesized that MGP expression is altered in chronic kidney disease. In a cohort of 571 healthy women, only dpucMGP (and not ucMGP or dpcMGP) was correlated with VC [41]. Likewise, in cohorts of CKD patients, the results regarding the possible association between MGP and VC produced controversial data [49,50,51,52], probably due to the fact that in these studies, ucMGP serum levels were quantified with a specific antibody that was not sensitive for the phosphorylation state of MGP.

UcMGP, an inactive form of MGP, maintains the calcium binding capacity and might be a novel VC biomarker.

### 3.5. Dephosphorylated, Uncarboxylated MGP: the Fully Inactive Circulating Form of MGP

To become fully active, after carboxylation, MGP must undergo a vitamin K-dependent phosphorylation [28]. Schurgers et al. developed specific antibodies to quantify all inactive forms of MGP (ucMGP, dpcMGP, dpucMGP), and found that phosphorylation of serine residues is the most crucial step in MGP activation. If MGP is carboxylated but not phosphorylated, it loses the ability to bind to BMP-2 or abrogate the osteoblast transformation of VSMCs [28,53]. Therefore, phosphorylated and non-phosphorylated forms of MGP, regardless of carboxylation status, have a different effect on VC. DpucMGP was accumulated in calcified vessels and circulating dpucMGP—probably reflecting vitamin K status— was associated with enhanced VC [18]. Histological findings of 100 carotid plaques of patients who underwent carotid endarterectomy suggested that plasma dpucMGP did not reflect ucMGP plaque levels [54]. A recent systematic review of 28 studies supported that dpucMGP, not ucMGP, is the fully inactivated form of MGP, unable to bind calcium or extracellular Gla matrix and therefore set free into circulation [53,55]. In contrast with ucMGP, which is not affected by vitamin K supplementation, circulating dpucMGP is a stronger indicator of vitamin K status and plays a pivotal role in the pathogenesis of VC [29,53,56,57,58]. In heart biopsies from patients deceased from dilated or ischemic cardiomyopathy, dpucMGP levels were significantly higher compared to the phosphorylated form [59]. Reduced dietary intake of vitamin K was associated with high serum dpucMGP levels and severe VC in various populations: healthy individuals [60], kidney transplant recipients [61], HD patients [62], diabetics [63], and heart failure patients [64].

### 3.6. DpucMGP and Renal Function

There is accumulating evidence showing that circulating dpucMGP is increased in CKD and further exacerbated in dialysis patients. Histological findings in 10 pre-dialysis and 24 dialysis-dependent children with CKD showed that, although all arteries had high levels of both cMGP and ucMGP, ucMGP was more expressed in the vessels of dialysis subjects [65]. Three studies showed that compared to healthy controls, HD patients had 4.5-fold [66], 5.6-fold [67], and 6.5-fold higher plasma dpucMGP levels [68], while another study reported on significantly lower serum of MGP in dialysis patients [69]. Similar results were published for pre-dialysis CKD patients. In a cohort of 107 patients in all 5 stages of CKD, Schurgers et al. found that dpucMGP augmented progressively with CKD stage and was strongly correlated with severity of aortic calcification. Furthermore, there was a strong inverse correlation between dpucMGP plasma levels and estimated glomerular filtration rate (eGFR) [53]. Roumeliotis et al. reported that circulating dpucMGP was increased in parallel to progression of CKD stages in a cohort of patients with diabetic nephropathy, distributed in all five stages of CKD. Moreover, plasma dpucMGP levels were correlated with albuminuria and proteinuria and inversely with eGFR. After adjustment for several risk factors, eGFR strongly predicted dpucMGP serum levels [70]. In agreement with these results, eGFR was a strong negative predictor of circulating dpucMGP in studies from the general population [71], in patients with advanced CKD [72,73], and in diabetics with normal [74] and mild or moderate renal impairment [75]. UcMGP plasma concentration was also associated with eGFR in a cohort of patients with stable CV disease [76]. Similarly, dpucMGP serum levels were increased according to disease severity in 137 CKD patients [73]. Compared to stage 4 CKD, patients undergoing maintenance HD had significantly higher plasma dpucMGP levels [72]. In a cohort of 83 patients with CKD stages 3, 4, and 5, plasma dpucMGP levels were 586, 870, and 1050 pmol/L respectively [77]. Similarly, in a cohort of 60 kidney transplant recipients, eGFR was a strong and independent predictor of plasma dpucMGP levels [61].

Wei et al. suggested that in a general Flemish population cohort, serum dpucMGP predicted deterioration of renal function, assessed by decrease in eGFR and increase in albuminuria [71]. The same group conducted a multi-ethnic population study and found that in 1166 Flemish and 714 South Africans, higher dpucMGP serum levels were associated with significant decrease in eGFR and increased risk for CKD progression [78]. Furthermore, in another general population study, high plasma dpucMGP levels were associated with increased risk for nephrolithiasis, revealing a direct causal relationship [79]. Renal resistive index (RRI), an ultrasound assessed index reflecting renal vascular damage, was associated with acute kidney injury (AKI), rapid eGFR decrease, mortality, and CV disease (CVD) [80]. A multicenter trial including 1035 community dwell subjects showed that plasma dpucMGP levels were independently correlated with RRI, after adjustment for several well-known CVD co-founders [81]. Rennenberg et al. enrolled 90 patients with moderate to severe hypertension and found that independently of renal function, the mean kidney fractional excretion of MGP was 12.8% [82]. A recent study investigated MGP expression in rats with 5/6 nephrectomy and reported that expression of MGP in the kidney was increased. In the same paper, the authors studied kidney biopsies from patients with nephrotic syndrome and found that eGFR was inversely associated with tubulointerstitial and glomerular MGP expression. Moreover, tubulointerstitial MGP expression was related with interstitial inflammation and fibrosis, tubular atrophy, and damage, independently of eGFR. Kaplan–Meier curves and multivariate Cox analysis showed that interstitial and tubular MGP overexpression was linked with a 40% reduction in eGFR and progression to end-stage renal disease (ESRD) with a Hazard ratio of 3.31. The authors of the study concluded that, both in animal models and humans, MGP is overexpressed in damaged kidneys and predicts deterioration of renal function [83]. Roumeliotis et al. measured dpucMGP serum levels in 55 patients with diabetic nephropathy in stages 1–4 and 11 HD subjects. After 7 years of follow-up, eGFR was re-estimated in CKD subjects, and in HD patients residual renal function was re-assessed. In CKD patients, circulating dpucMGP was inversely associated with both baseline and, after the follow-up time, eGFR, while in HD subjects residual renal function (RRF) was correlated with decreased serum dpucMGP levels. Moreover, multivariate regression analysis showed that after adjustment for several co-founders, dpucMGP predicted both new eGFR value and eGFR decline [84]. 

There is a growing body of evidence supporting that CKD patients have a high prevalence of subclinical vitamin K status resulting in subsequent low serum levels of all VKDPs [85,86,87]. The data suggest that MGP, besides a powerful natural VC inhibitor, exerts a renoprotective role [88].

The fully inactive form of MGP, dpucMGP, which probably reflects vitamin K status, is tightly associated with deterioration of renal function.

## 4. DpucMGP and Arterial Calcification/Stiffness

Several investigators reported association of serum dpucMGP levels with well-known traditional co-founders for atherosclerosis (such as dyslipidemia, impaired glucose tolerance, obesity, inflammation, advanced age) [57,61,66,70,89]. The possible association between plasma dpucMGP and VC was repeatedly investigated in populations with high atherogenic status, such as elderly, uremic, HD, diabetics, atherosclerotic, and hypertensive patients. Table 1 summarizes the associations between circulating levels of MGP forms and aortic stiffness/arterial calcification scores.

### 4.1. General Population

Several investigators have highlighted the tight linkage between circulating dpucMGP and arterial stiffness/calcification in the general population. Mayer et al. studied 1087 subjects from the general population and reported that dpucMGP levels were independently associated with aortic and femoro–popliteal PWV [93]. In 244 healthy post-menopausal women, serum dpucMGP levels were strongly correlated with cIMT, carotid–femoral PWV, and endothelial dysfunction score [92]. A multicenter family-based trial enrolled 1001 subjects and found that even after adjustment for renal function, diabetes, and CVD, serum dpucMGP levels were significantly and independently correlated with aortic PWV [91]. In 571 women from general population, only circulating dpucMGP (and not ucMGP or dpcMGP) was closely correlated with the presence of CAC [41]. In a cohort of patients aged <55 years, receiving vitamin K antagonist but without any other risk for CVD, dpucMGP serum levels were significantly correlated with calcification of the femoral artery [95]. After 3 years of vitamin K1 supplementation (500 μg/day), dpucMGP plasma levels were significantly reduced, but still no association with the change in CAC score was revealed [90]. The results of this study were in contrast with findings of other studies that showed increased circulating dpucMGP levels in various cohorts of patients with conditions characterized by enhanced VC. The authors proposed several reasons explaining this discrepancy of their results: firstly, the population of the study consisted of healthy old adults with no history of calcification, secondly, calcification was assessed directly by CAC score, and thirdly, circulating dpucMGP might reflect VC status that is not associated with the atherosclerosis process.

### 4.2. Patients with Diabetes Mellitus

In a multi-ethnic group of 66 patients with type 2 diabetes mellitus (T2DM), plasma dpucMGP levels were strongly correlated with carotid–femoral PWV, a marker of large artery stiffness [74]. Similarly, a cross-sectional study including 198 T2DM patients with normal or mildly impaired renal function showed that only serum levels of dpucMGP (and not ucMGP) were independently associated with peripheral VC (below-knee calcifications assessed by computed tomography) [75]. In disagreement with these results, in a cohort of 142 T2DM patients with various degrees of renal impairment, circulating dpucMGP was not correlated with cIMT [70]. In this study, atherosclerosis was assessed by cIMT. In agreement with previous studies, the authors hypothesized that dpucMGP might reflect certain processes of VC not associated directly with the atherosclerosis process. A recent systematic review investigating the possible association of MGP fractions and VC included one study on diabetics and four on CKD patients, and reported that dpucMGP might be a novel VC marker in these populations [55].

### 4.3. Patients with Hypertension

Chirinos et al. showed that carotid–femoral PWV was independently associated with plasma dpucMGP concentration in a group of 199 hypertensive subjects [96].

### 4.4. CKD and HD Patients

Circulating dpucMGP was associated with aortic calcification score in a cohort of 107 patients with various stages of CKD (2–5) [53]. In a similar population of 83 CKD patients at stages 3, 4, and 5, Thamratnopkoon et al. showed that dpucMGP serum levels were significantly associated with the VC marker—abdominal aortic calcification score (AAC), but not with vascular stiffness—assessed by cardio–ankle vascular index and PWV [77]. Similarly, in 137 patients with various degrees of kidney function, including patients with normal renal function and CKD stages 2–5, after adjustment for several confounders, dpucMGP plasma level was a strong independent predictor of carotid–femoral PWV [73]. In disagreement with these results, Roumeliotis et al. found no association between serum dpucMGP levels and cIMT in a cohort of 142 patients with DN and CKD stage 1–5 [70]. Likewise, in a cohort of 37 African American HD patients, after adjustment for age, sex, diabetes, body mass index (BMI), blood pressure, and CVD, serum dpucMGP concentration was tightly correlated with PWV and endothelial dysfunction [67]. An observational study on 136 HD patients reported that circulating dpucMGP was independently associated with AAC [57], and another study showed strong association between dpucMGP serum levels and calcification of the abdominal aorta—assessed by the aortic calcification severity score—in a group of 50 stable, chronic HD patients [94]. In disagreement with these results, Schlieper et al. reported no association between circulating dpucMGP or dpcMGP and cIMT or PWV in a cohort of 188 stable HD patients [68].

Data from studies on various populations, including healthy subjects and patients with high atherogenic status, suggest that circulating dpucMGP might be a novel early marker of VC.

## 5. DpucMGP and Mortality/CV Events

Since there is accumulating data suggesting that dpucMGP might be a novel marker of VC, several studies have explored the possible association of dpucMGP with mortality and CV events in various populations. An overview of association between circulating levels of MGP forms and adverse events is presented in Table 2.

### 5.1. General Population

A study enrolled 577 adults from general population, aged >55 years, of the Longitudinal Aging Study Amsterdam study with no history of previous CVD. At baseline, plasma levels of both dpucMGP and dpcMGP were measured. All patients were followed for a mean period of 5.6 ± 1.2 years and 40 incident cases of CVD were identified. After adjustment for several confounders, there was a more than 2-fold risk for CVD in the highest tertile of dpucMGP group (HR = 2.69, 95% CI 1.09–6.62) compared to the lowest tertile, while dpcMGP failed to show any association with CVD [97]. Similar results were reported by Liu et al. in a Mendelian randomization analysis study. In 2318 community-dwelling Flemish subjects, dpucMGP serum levels were quantified in baseline. After a follow-up period of 14.1 years, increased serum concentration of dpucMGP was an independent predictor of total, non-cancer, and CV mortality. Surprisingly, higher dpucMGP plasma levels were associated with lower incidence of coronary events. The association between serum dpucMGP and non-cancer mortality was probably causal [98]. In disagreement with these results, another prospective study showed that after a mean follow-up period of 11.5 years, serum dpucMGP levels were not associated with the incidence of stroke or CAD in 1406 subjects from general population, aged >49 years [99].

### 5.2. Patients with Diabetes Mellitus

Dalmeijer et al. measured serum levels of MGP forms (dpucMGP, dpcMGP, ucMGP) in 518 T2DM patients. After a median follow-up of 11.2 years, only high serum levels of dpucMGP were associated with increased risk for CVD, peripheral artery disease (PAD), and heart failure, but not with CAD or stroke. There was no association between any of the study’s end-points and serum dpcMGP or ucMGP [63]. In a cohort of 198 T2DM patients with normal or mild impairment of renal function, only serum dpucMGP (but not ucMGP) was a strong and independent predictor for PAD [75]. Circulating dpucMGP was an independent predictor for overall mortality and fatal/non-fatal CV events in 67 T2DM patients with normal, mild, moderate, and severe renal impairment [100].

### 5.3. CKD and HD Patients

Plasma dpucMGP concentration was measured in a cohort of 107 patients at CKD stages 2–5, During a 802 ± 311 days follow-up, 34 subjects died, 20 from CVD. Kaplan–Meier curves showed that high serum dpucMGP > 921 pM (median value) was associated with increased overall mortality (*p* = 0.006, log-rank test). This association was lost after adjusting for age and severity of VC [53]. In a prospective study, Roumeliotis et al. enrolled 57 patients with established DN, distributed across all 5 stages of CKD (stage 1 and 2: 10, stage 3: 26, stage 4: 10, stage 5: 11), and 10 T2DM with normal renal function, the control group. DpucMGP serum levels were assessed at enrolment. All patients were followed for 7 years, with end points all-cause/CV mortality and non-fatal CV event. Kaplan–Meier curves showed that circulating dpucMGP > 646 pM (median value) was associated with all-cause, CV mortality, and CV events (*p* = 0.011, *p* = 0.008, and *p* = 0.019 respectively, log-rank test). After adjustment for several established risk factors for mortality and CVD (age, sex, BMI, history of CVD, smoking, duration of hypertension and T2DM, dyslipidemia, glycated hemoglobin), multivariate Cox analysis showed that high serum dpucMGP > 646pM was associated with higher all-cause mortality (HR 2.97, 95% CI = 1.27–6.95, *p* = 0.012), CV mortality (HR 5.49, 95% CI = 1.85–16.33, *p* = 0.002), and non-fatal CV events (HR 2.07, 95% CI = 1.00–4.20, *p* = 0.047) compared to patients in the low dpucMGP group [100]. Similarly, in a cohort of 518 kidney transplant recipients with CKD, increased plasma levels of dpucMGP were associated with a three-fold higher overall mortality risk and a more than two-fold risk for incidence of transplant failure. After adjustment for several confounders, the association between circulating dpucMGP and higher mortality risk persisted and with transplant failure was lost [101]. 

Both dephosphorylated forms of MGP (dpucMGP and dpcMGP) were assessed in a cohort of 188 stable, maintenance HD patients, followed for 3 years. Both Kaplan–Meier curves and multivariate Cox analyses adjusted for age showed that low dpcMGP < 6139 pmol/L was associated with overall mortality (HR 2.31, 95% CI = 1.2–4.4, *p* = 0.01) and CV mortality (HR 2.94, 95% CI = 1.4–6.3, *p* = 0.006). Although Kaplan–Meier curves showed that dpucMGP was marginally not associated with overall (*p* = 0.08, log-rank test) and CV mortality (*p* = 0.09, log-rank test), univariate Cox analysis showed that low serum levels of dpucMGP < 442 pmol/L were associated with overall mortality (HR 1.71, 95% CI = 0.92–3.17, *p* = 0.09), and CV mortality (HR 1.83, 95% CI = 0.90–3.70, *p* = 0.09) [68].

### 5.4. Patients with High CVD Risk and Heart Failure

Ueland et al. showed that only circulating dpucMGP (and not dpcMGP) was strongly and independently associated with deterioration of heart failure and overall mortality in a cohort of 147 patients with symptomatic, severe, calcific aortic stenosis [89]. In agreement with these results, a recent study reported that high plasma dpucMGP levels were associated with deterioration of heart function (diastolic left ventricular dysfunction) in both epidemiological and histological findings in the general population, as well as patients with heart failure [59]. In 179 patients with chronic heart failure, high serum dpucMGP (and not dpcMGP) levels were strongly and independently associated with death from deterioration of heart failure [64]. The multi-center ASTRONOMER trial (aortic stenosis observation: measuring effects of rosuvastatin), included 215 patients aged 18–82 years with mild or moderate aortic stenosis, and reported that high serum dpMGP levels were independent predictors of disease progression, especially in younger subjects [102]. Mayer et al. conducted a prospective cohort trial to investigate the possible predictive value of dpucMGP for mortality in subjects with stable vascular disease. For a median of 5.6 years, 799 patients with history of myocardial infraction (MI), stroke, or CAD were followed. In multivariate Cox regression analysis, it was shown that patients in the highest dpucMGP tertile (dpucMGP over 977 pmol/L) had a significantly increased risk for CV and overall mortality (HR 1.88, 95% CI = 1.22–2.90 and HR 1.89, 95% CI = 1.32–2.72, respectively). Corresponding HR for serum dpcMGP were 1.76, (95% CI = 1.18–2.61) and 1.79 (95% CI = 1.12–2.57). Low ucMGP plasma levels < 2825 nmol/L were associated with high risk for overall mortality (HR 1.43, 95% CI =1.01–2.03) and CV mortality (HR 1.33, 95% CI =1.01–2.01). The authors concluded that, since the data regarding the association of dpcMGP and ucMGP and mortality/CVD remains controversial, only circulating dpucMGP should be assessed as a novel potential CV risk factor [103,104]. An observational, multicenter, prospective trial enrolled 833 outpatients with stable CAD and followed them for a median of 6 years. Low plasma ucMGP levels were associated with higher risk for overall mortality and CV events [42]. A similar cross-sectional study in 839 outpatients with stable CVD reported that increased plasma ucMGP was associated with lower incidence of mitral annular calcification in non-diabetics and higher incidence of mitral annular calcification in diabetics [52]. Among 40 patients scheduled for orthopedic or abdominal surgery, serum dpucMGP levels were significantly higher in those with previous history of CVD compared to those with no record, while dpcMGP levels were not significantly different among groups. Post-operatively, only serum dpucMGP (and not dpcMGP) levels were significantly increased in the group with history of CVD [105]. A recent meta-analysis including 7 studies and 3301 patients reported that circulating inactive dpucMGP was a strong independent predictor of overall and CV mortality, but not CVD [106]. 

Of all inactive forms of MGP, circulating dpucMGP might be considered as a novel strong risk factor for mortality and cardiovascular events.

## 6. MGP Gene Polymorphisms, VC, Mortality and CVD 

The study by Luo et al. where knockout MGP mice (MGP −/−) had normal phenotype at birth, but after 6–8 weeks died from accelerated VC, led investigators to explore the possible association between gene MGP polymorphisms and VC [14]. Moreover, Keutel syndrome, a rare autosomal inherited condition characterized by enhanced chondrogenesis, is caused by mutations in the MGP gene [15,107,108]. The most frequent single nucleotide polymorphisms of MGP are T-138C (rs 1800802), located in the promoter region, and G-7A (rs 1800801), located in the5’-untranslated region, and both are associated with arterial calcification [109].

Compared to the C allele, the T allele of the MGP T-138C polymorphism consistently conferred an increased promoter activity [110]. However, whether the T-138C polymorphism has an effect on MGP serum levels remains controversial. Several studies report no significant changes of serum MGP levels among the 3 different genotypes (TT, TC, CC) [70,111,112,113]. Although T-138C was not associated with MGP plasma levels in CKD patients, CC homozygotes had higher MGP levels (but without reaching statistical significance), suggesting that TT genotype might be related to accelerated VC [112]. Furthermore, a study of 108 autopsies reported that the T allele consistently conferred an increased promoter activity, but neither of the two alleles was associated with abdominal aorta calcification [114]. Likewise, in patients without VC, the C allele was correlated with higher MGP in serum [115]. In disagreement with these results, Wang et al. found that in ESRD patients, the T allele was four times less active compared to C and therefore, the CC genotype might be associated with increased expression of MGP and abrogated VC [116]. Similarly, Farzaneh-Far et al. showed that compared to T, the C allele was associated with increased MGP transcription, and subsequently higher circulating MGP and lower calcification status [117]. Subsequently, plasma MGP levels of -138TT homozygotes are 30% lower compared to CC genotypes [117,118]. In line with these results, in a cohort of 142 T2DM patients with various degrees of DN, Roumeliotis et al. showed that TT genotypes were associated with accelerated VC—higher cIMT values compared to TC and CC, independently of serum dpucMGP levels [70]. Likewise, Yoshikawa et al. reported that compared to TC and TT, CC homozygotes presented lower degree of VC in HD patients independently of serum MGP levels [111]. In men with normal kidney function, CC homozygotes presented a lower coronary calcification status compared to TT subjects [119]. Two studies reported that the distribution of MGP T-138C genotypes was significantly different in HD patients compared to healthy controls. Roumeliotis et al. included 112 patients with DN and CKD 1-4 or ESRD on HD and 40 T2DM subjects with normal renal function—controls—and found an excess of TT genotype in the HD group, compared to both controls and patients with CKD stage 1–4. The high frequency of TT homozygotes in the HD group was even more pronounced when TC and CC genotypes were grouped together. After a 7 year follow-up, multivariate Cox regression analysis showed that, compared to TC/CC genotypes, TT homozygotes had a significantly higher risk for overall and CV mortality (HR 4.67, 95% CI = 1.37–15.94, *p* = 0.01, and HR 5.07, 95% CI = 1.07–24.09, *p* = 0.04, respectively [70]. Similarly, Brancaccio et al. reported that the frequency of the T allele was significantly higher in HD patients compared to healthy controls. After 1 year of follow-up, 17 of the 99 HD patients died from CVD. Sixteen of them (94.1%) were TT homozygotes [118]. To investigate the possible confounders involved in the pathogenesis of CAD even in its early stages, the CARDIA study (Coronary Artery Risk Development in Young Adults) was a large prospective trial with 5116 subjects, aged 18–30. Data from this study reported that the T-138C polymorphism was not associated with CAD development, probably due to the young age of the participants [120].

Several observational studies have reported association of MGPG-7A polymorphism with VC and CVD: calcification in aorta and coronary arteries [115], atheromatic plaque and a nearly 4-fold risk for MI [110], ischemic atheroembolic stroke [121,122,123], and acute coronary episode [124,125]. In a cohort of 438 adults aged >60 years from the general population, G-7A polymorphism was an independent predictor of plasma dpucMGP levels. The major allele homozygotes for G-7A polymorphism had significantly increased plasma dpucMGP concentration, compared to heterozygotes and minor allele homozygotes. However, no association was found between T-138C and plasma dpucMGP [90]. Moreover, in 296 community-dwelling adults, aged 50–79, G-7A, but not T-138C, was associated with development of osteoporosis and aortic calcification [126]. In disagreement with the previous results, in a cohort of 182 patients who underwent coronary angiography (112 with diagnosed CAD and 70 with no critical stenosis), MGP G-7A and T-138C were not associated with coronary artery stenosis [113,127]. A recent meta-analysis included 23 studies, 5280 patients, and 5773 controls to explore the possible association of MGP polymorphisms and VC. The authors concluded that, although MGP G-7A was associated with a 1.5-fold risk for developing VC and atherosclerosis, no association was found between T-138C and these end-points [128]. Moreover, the frequency of the G allele of MGP-7 polymorphism was significantly lower in HD subjects and patients with CKD stage 3 compared to age- and gender- matched healthy controls [118].

MGP G-7A and T-138C polymorphisms might genetically predispose to early and enhanced VC. Future trials in large cohorts are needed in order to elucidate this possible association.

## 7. Vitamin K Affects Activation of MGP

There is accumulating data suggesting that vitamin K (especially K2) is inversely correlated with VC and CVD. The large epidemiological Rotterdam study of 4807 subjects free of CVD at baseline showed that dietary intake of vitamin K2 was inversely associated with aortic calcification, all-cause, and CAD mortality, whereas K1 was not linked with any of these outcomes [129]. In a cohort of 564 post-menopausal women, Beulens et al. reported that only high dietary K2 intake, but not K1, was associated with significantly decreased coronary calcification [130]. The findings of this study were in agreement with those reported by the epidemiological Rotterdam study. The authors hypothesized that the different effects of vitamin K1 and K2 on VC might be explained by the different metabolic pathways of these two forms, resulting in different concentrations in plasma lipoproteins. K1 is predominantly transported in the liver and influences the activation of blood coagulation factors, while K2 is equally found in both liver and extrahepatic tissues such as the vasculature, and subsequently is more effectively involved in MGP activation. The European Prevalence of Infection in Intensive Care (EPIC) Study enrolled 16,057 women aged 49–70 years, with no previous history of CVD. In this cohort, after adjustment for well-known confounders, high dietary intake of vitamin K2 (especially subtypes MK-7, MK-8, MK-9) was negatively associated with risk of CAD [131]. However, a recent meta-analysis of 4 trials with 48,713 participants showed no association between dietary intake of vitamin K and K1, K2 subtypes, and all-cause/CV mortality [106], while a systematic review highlighted the need for further large placebo-controlled trials, since data on the effect of vitamin K on MGP species and mortality and CVD are extremely limited [132].

CKD patients carry a heavy CV burden that is gradually augmented with disease progression and is further increased in HD. Several investigators highlighted that uremic patients [61,87,133] present subclinical vitamin K deficiency, which becomes more pronounced in HD [85,86,134]. Several investigators repeatedly showed that of all circulating MGP species (dpucMGP, ucMGP, dpcMGP), only dpucMGP circulating levels were strongly and inversely correlated with plasma concentration of vitamin K in various populations -including healthy subjects [60,61,68] -, and therefore only circulating dpucMGP reflects vitamin K status. Moreover, animal studies showed that vitamin K2 (MK-7) supplementation triggers MGP expression, decreases circulating dpucMGP, and abrogates VC [38,135,136]. Therefore, several investigators aimed to explore whether vitamin K administration could potentially normalize serum dpucMGP levels and subsequently protect from CV events.

## 8. Effect of Vitamin K Supplementation on MGP Forms in Human Interventional Studies

Table 3 shows an overview of vitamin K effects on circulating levels of MGP forms.

### 8.1. General Population

To investigate the possible effect of MK-7 intake on circulating forms of MGP, Dalmeijer et al. recruited 60 subjects from the general population, aged 40–65, and randomized them to treatment with daily oral intake of either 180 μg or 360 μg of MK-7 or placebo for 12 weeks. At baseline, after 1 month and at the end of the study, plasma levels of dpucMGP, dpcMGP, and ucMGP were assessed by ELISA. After 3 months treatment, circulating dpucMGP was dose-dependently reduced in the 180 and 360 μg supplementation group by 31% and 46% respectively, whereas it remained unchanged in the placebo group. There were no significant changes in plasma levels of dpcMGP and ucMGP in all groups. The authors of the study concluded that only circulating dpucMGP reflects vitamin K levels, and that it might be a novel therapeutic target for reducing CVD [137]. A group of 44 healthy subjects, aged 18–45 years, were divided to receive daily placebo, low (below the recommended daily dose, 75 μg/day), or high dose (>75 μg/day) of MK-7 for 12 weeks. Circulating dpucMGP was increased in the placebo group and slightly altered in the low MK-7 group, whereas it was significantly decreased in the high MK-7 group. At the end of the study only MK-7, but not K1, plasma concentrations were strongly and inversely correlated with changes in dpucMGP circulating levels. The authors concluded that daily oral intake of high-dose MK-7 improves carboxylation status of MGP, and subsequently might protect against CVD [138]. Two randomized placebo-controlled trials included 55 healthy children aged 6–10 years and allocated them to either 45 μg/day of MK-7 or placebo for 8 weeks, while 69 healthy adults aged 20–40 years were randomized to receive 90 μg/day of MK-7/day or placebo for 7 weeks. In healthy children and adults, MK-7 supplementation was associated with a 38% and 36% decrease in serum dpucMGP levels respectively [139]. In a double-blind, placebo-controlled trial, 244 healthy, post-menopausal women were randomized to MK-7 or placebo for 3 years. Compared to placebo, MK-7 treatment resulted in a 50% decrease of circulating dpucMGP, accompanied by a significant improvement of arterial stiffness [92]. A cohort of 388 healthy men and post-menopausal women were allocated to receive daily a multivitamin enhanced with 500 μg K1 or multivitamin alone. After 3 years of treatment, K1 supplementation slowed VC progression, independently of changes in serum MGP. MGP serum levels increased in the K1 group and decreased in the control group but were not associated with VC progression. The authors of the study hypothesized that the findings of their study might be explained by the fact that the non-carboxylated and de-phoshorylated forms of MGP were not quantified [140]. The same group of investigators showed that in 374 community–dwelling adults, aged 60–80 years, plasma dpucMGP levels were significantly reduced in those treated with 500 μg/day of K1 for 3 years, compared to those who were not. There was no association between dpucMGP plasma levels at baseline and at the end of the study with VC [90]. Eighteen healthy subjects received vitamin K antagonists for 4 weeks, and after that for 6 subsequent weeks were treated with combined therapy of vitamin K antagonist and MK-7 at increasing daily doses of 10, 20, and 45 μg. Vitamin K antagonist treatment caused a significant increase in dpucMGP serum levels. Even the highest MK-7 could not reduce circulating dpucMGP [141].

### 8.2. CKD and HD Patients

A prospective clinical trial in a cohort of 50 maintenance HD patients reported that, after 4 weeks of treatment with 360 μg/day MK-7, dpucMGP serum levels were decreased by 86%, with the lowest reduction rate presented in diabetics [94]. Similarly, in a randomized study, 53 stable HD patients were treated with 45, 135, or 360 μg/day of MK-7 for 6 weeks, and after the study period, circulating dpucMGP showed a dose- and time-dependent reduction rate [66]. Even the highest MK-7 dose could not normalize serum dpucMGP levels (93% reduction). Likewise, in a dose-finding study, Caluwe et al. treated 200 HD patients with 360, 720, or 1080 μg MK-7 thrice weekly for 2 months. At baseline, dpucMGP plasma levels were inversely correlated with MK-7 dietary intake, but not with K1. After the study period, in the three groups, dpucMGP was reduced by 17, 33, and 46% respectively [62]. In a small cohort of 17 HD patients, daily oral administration of 135 μg MK-7 for 6 weeks resulted in a 27% decrease of circulating dpucMGP, whereas dpcMGP serum levels remained unchanged [68]. Moreover, stopping vitamin K antagonist treatment in 7 stable HD patients was linked with a rapid time–effect decrease in serum dpucMGP levels. Within 5 days after stopping the treatment, circulating dpucMGP was decreased by at least 40% [142]. Kurnatowska et al. randomized 42 CKD pre-dialysis patients to receiving daily 90 μg MK7 with 10 μg vitamin D or 10 μg vitamin D for 270 days. After the study period, compared to patients treated only with vitamin D, the vitamin K + D group resulted in a significant 19% reduction of plasma dpucMGP levels, accompanied by a slower progression of atherosclerosis [143]. In a similar study, 38 pre-dialysis CKD patients were supplemented for 270 days with daily 90 μg MK7 and 10 μg vitamin D, or 10 μg vitamin D alone. MK-7 supplementation resulted in a 10.7% reduction of circulating dpucMGP [72].

### 8.3. Patients with High CVD Risk and Heart Failure

Brandenburg et al. allocated 72 patients with aortic valve calcification and normal renal function to either 2 mg of vitamin K1 or placebo once daily for 12 months. After the treatment period, the vitamin K group presented significantly decreased serum dpucMGP levels (−45%) and slower progression of cardiac valve calcification compared to the placebo group [144]. In disagreement with these results, all MGP forms (including dpucMGP) were refractory to vitamin K1 supplementation (10 μg/day for 3 months) in a male 21 year old patient with Keutel syndrome [108].

### 8.4. Ongoing Clinical Trials

Several ongoing, randomized, placebo-controlled trials are ongoing and may elucidate the possible effect of vitamin K supplementation on MGP species’ serum levels and hard end-points [145]: the inhibiting the progression of arterial calcification with vitamin K in hemodialysis patients trial (iPACK-HD) [146], the vitamin K1 to slow VC in HD subjects study (VitaVasK) [147], the bicuspid aortic valve stenosis and the effect of vitamin K2 on calcification trial (BASIK2) [148], and the menaquinone-7 supplementation to reduce vascular calcification in patients with coronary artery disease trial (VitaK-CAC) [149].

Supplementation of vitamin K2 (especially MK-7) seems to upregulate MGP activation and decrease the circulating inactive forms of MGP. Therefore, K2 intake might be beneficial and protect against future CV events in both healthy populations and patients that carry a heavy CV burden.

## 9. Conclusions

Vascular calcification is not a passive, degenerative, untreatable disease, but an active process in which proteins and molecules are involved. MGP is the most powerful natural calcification inhibitor found in the human body, and is tightly associated with all types of calcification, mortality, and cardiovascular disease. Exogenous supplementation of vitamin K might upregulate its function, reduce calcification, and protect against mortality and cardiovascular disease. The sensitivity of dpcMGP assay is less compared to that of the dpucMGP assay. In future trials, among all inactive MGP forms, ucMGP maintains calcium-binding capacity and might be a VC marker, whereas dp-ucMGP might be considered a novel risk factor for mortality and CVD. 

## Figures and Tables

**Table 1:** Overview of associations between circulating levels of MGP forms and aortic stiffness/arterial calcification scores.

Population, Age, Study, Design	MGP Form	Calcification Dcore	Result
**General Population**			
438 general population adults, 68 y, [90], cross-sectional	DpcMGP (pmol/L)	CAC	-
571 general population women, 57.3 y, [41], observational, prospective	DpucMGP (pmol/L)	CAC	↑
	UcMGP (nmol/L)		-
	DpcMGP (pmol/L)		-
1001 general population subjects, 46.5 y, [91], multicenter, family-based, cross-sectional	DpucMGP (pmol/L)	Aortic PWV	↑
244 healthy post-menopausal women, 59.5 y, [92], sross-sectional	DpucMGP (pmol/L)	cIMT	↑
		Carotid–femoral PWV	↑
		Endothelial dysfunction score	↑
1087 general population subjects, 54.8 y, [93], cross-sectional	DpucMGP (pmol/L)	Femoro–popliteal PWV	↑
**CKD and HD Patients**			
120 HD patients, 61 y, [44], cross-sectional, multicenter	UcMGP (nmol/L)	Aortic augmentation index	↓
		PWV	-
61 HD children, 13.4 y, [47], cross-sectional	UcMGP (nmol/L)	cIMT	-
		Carotid–femoral PWV	--
		Aortic augmentation index	-
		CAC	-
40 HD patients, 67 y, [45], cross-sectional	UcMGP (nmol/L)	CAC	↓
107 patients with CKD stages 2–5, 67 y, [53], cross-sectional, prospective	DpucMGP (pmol/L)	Aortic calcification score	↑
188 HD patients,59 y, [68], cross-sectional	DpucMGP (pmol/L)	cIMT	-
		PWV	-
	DpcMGP (pmol/L)	cIMT	-
		PWV	-
136 HD patients, 74 y, [57], cross-sectional	DpucMGP (pmol/L)	Abdominal aortic calcification score	↑
83 patients with CKD stages 3–5, 62.9 y, [77], cross-sectional	DpucMGP (pmol/L)	Abdominal aortic calcification	↑
		Cardio–ankle vascular index	-
		PWV	-
50 HD patients, 71.5 y, [94], cross-sectional	DpucMGP (pmol/L)	Abdominal aortic calcification score	↑
137 patients with various degrees of CKD, 60.7 y, [73], cross-sectional	DpucMGP (pmol/L)	Carotid–femoral PWV	↑
37 HD patients with CKD stages 2–5, 47.7 y, [67], cross-sectional	DpucMGP (pmol/L)	Carotid–femoral PWV	↑
		Brachial artery flow-mediated dilation	↑
**High CVD Risk Patients**			
191 aortic valve disease patients, 71 y, [49], observational	UcMGP (nmol/L)	Aortic valve calcification	-
19 subjects treated with vitamin K antagonist, 48 y, [95], observational	DpcMGP (pmol/L)	Femoral artery calcification	↑

DpucMGP: dephosphorylated uncarboxylated matrix Gla protein; dpcMGP: dephoshorylated carboxylated matrix Gla protein; ucMGP: uncarboxylated matrix Gla protein; dpMGP: dephosphorylated matrix Gla protein; PWV: pulse wave velocity; CAC: coronary artery calcium score; cIMT: carotid intima-media thickness; CVD: cardiovascular disease; CKD: chronic kidney disease; HD: hemodialysis, T2DM: type 2 diabetes mellitus. - = no statistically significant difference, ↑ = statistically significant positive association, ↓ = statistically significant negative association.

**Table 2:** Overview of associations between circulating levels of MGP forms and adverse events.

Population, Age, Study, Design	MGP Form	End-Point	Result
**General Population**			
1406 general population subjects, >49 y, [99], prospective, obseravtional	DpucMGP (pmol/L)	Stroke	-
		CHD	-
	DpucMGP (pmol/L)		↑
577 general population subjects, >55 y, [97], cross-sectional	DpcMGP (pmol/L)	CV event	-
2318 general population subjects, 43.5 y, [98], cross-sectional	DpucMGP (pmol/L)	Overall mortality	↑
		Non-cancer mortality	↑
		CVD mortality	↑
		Coronary events	↓
1054 general population subjects, 49.5 y, [59], population-based, cross-sectional	DpucMGP (μg/L)	Left ventricular dysfunction	↑
**Patients with diabetes**			
	DpucMGP (pmol/L)	CV events	↑
		PAD	↑
		HF	↑
		CHD	-
		Stroke	-
	DpcMGP (pmol/L)	CV events	-
		PAD	-
		HF	-
		CHD	-
		Stroke	-
518 T2DM patients, 58.1 y, [63], prospective, observational	UcMGP (nmol/L)	CV events	-
		PAD	-
		HF	-
		CHD	-
		Stroke	-
	DpucMGP (pmol/L)		↑
198 T2DM patients with various degrees of CKD, 64 y, [75], cross-sectional	UcMGP (nmol/L)	PAD	-
**CKD and HD patients**			
107 CKD patients, stages 2–5, 67 y, [53], prospective, cross-sectional	DpucMGP (pmol/L)	Overall mortality	↑
	DpucMGP (pmol/L)	Overall mortality	↓
		CV mortality	↓
188 HD patients, 59 y, [68],	DpcMGP (pmol/L)	Overall mortality	↓
cross-sectional		CV mortality	↓
518 kidney transplant recipients, 51 y, [101], observational with longitudinal design	DpucMGP (pmol/L)	Overall mortality	↑
		Transplant failure	↑
57 T2DM patients in CKD stages 1–5 and 10 T2DM controls, 68.6 y, [100], prospective, cross-sectional	DpucMGP (pmol/L)	Overall mortality	↑
		CV mortality	↑
		CV events	↑
**High CVD risk and HF patients**			
	DpucMGP (pmol/L)	Overall mortality	↑
		HF	↑
147 aortic stenosis patients, 74 y, [89], cross-sectional	DpcMGP (pmol/L)	Overall mortality	-
		HF	-
833 CHD patients, 67 y, [42], observational, multicenter	UcMGP (nmol/L)	Overall mortality	↓
		CV events	↓
615 CVD non-diabetic patients, 68 y, [52], cross-sectional			↓
221 CVD, diabetic patients, 68 y, [52], cross-sectional	UcMGP (nmol/L)	Mitral annular calcification	↑
	DpucMGP (pmol/L)		↑
179 HF patients, 56 y, [64], cross-sectional	DpcMGP (pmol/L)	HF mortality	-
215 aortic stenosis patients, 18–82 y, [102], cross-sectional	DpMGP	AS progression	↑
	(nmol/L)		
	DpucMGP (pmol/L)	Overall mortality	↑
		CV mortality	↑
	DpcMGP (pmol/L)	Overall mortality	↑
		CV mortality	↑
799 CVD patients, 65.1 y, [103,104], prospective, cross-sectional, multicenter	UcMGP (nmol/L)	Overall mortality	↓
		CV mortality	↓

DpucMGP: dephosphorylated uncarboxylated matrix Gla protein; dpcMGP: dephoshorylated carboxylated matrix Gla protein; ucMGP: uncarboxylated matrix Gla protein; dpMGP: dephosphorylated matrix Gla protein; CV: cardiovascular; CVD: cardiovascular disease; CHD: coronary heart disease; PAD: peripheral artery disease; HF: heart failure; AS: aortic stenosis; CKD: chronic kidney disease; HD: hemodialysis; T2DM: type 2 diabetes mellitus. - = no statistically significant difference, ↑ = statistically significant positive association, ↓ = statistically significant negative association.

**Table 3:** Overview of vitamin K effects on circulating levels of MGP forms.

Population, Age, Study, Design	Study Groups	Intervention Time	End-Points	Result
**General Population**				
	500 mg/day K1	3 years	MGP (ng/mL)	↑ 3.5%
388 healthy men and postmenopausal women, 68 y, [140], randomized, double-blind, placebo-controlled	placebo			↓ 4%
	500 mg/day K1 + 10 μg vitamin D			↓ 80%
374 general population subjects, 60–80 y, [90], randomized, placebo-controlled	Placebo + 10 μg vitamin D	3 years	UcMGP (pmol/L)	↓ 4%
			DpucMGP (pmol/L)	↓ 31%
			DpcMGP (pmol/L)	-	
	180 μg/day MK-7		UcMGP (nmol/L)	-
			DpucMGP (pmol/L)	↓ 46%
			DpcMGP (pmol/L)	-
	360 μg/day MK-7		UcMGP (nmol/L)	-
			DpucMGP (pmol/L)	-
60 general population subjects, 40–65 y, [137],			DpcMGP (pmol/L)	-
randomized, double-blind, placebo-controlled	placebo	12 weeks	UcMGP (nmol/L)	-
	10 μg/day MK-7			↓ 12.1%
	20 μg/day MK-7			↓ 10.9%
	45 μg/day MK-7			↓ 12.3%
	90 μg/day MK-7			↓ 33.6%
	180 μg/day MK-7			↓ 39.7%
	360 μg/day MK-7			↓ 56%
44 general population subjects, 18–45 y, [138], randomized, double-blind, placebo-controlled	placebo	3 months	DpucMGP (pmol/L)	↑ 16.8%
	10 μg/day MK-7			-
	20 μg/day MK-7	6 weeks	DpucMGP (pmol/L)	-
18 healthy subjects treated with VKA for 4 weeks, 29 y, [141], observational	45 μg/day MK-7			-
	45 μg/day MK-7			↓ 38%
42 healthy children, 6–10 y, [139], randomized, placebo-controlled	placebo	2 months	DpucMGP (pmol/L)	-
	90 μg/day MK-7			↓ 36%
69 healthy subjects, 20–40 y, [139], randomized, placebo-controlled	placebo	7 weeks	DpucMGP (pmol/L)	-
244 healthy, postmenopausal women, 59.5 y, [92], randomized, double-blind, placebo-controlled	180 μg/day MK-7			↓ 32%
	placebo	3 years	DpucMGP (pmol/L)	↑ 22%
**CKD and HD patients**				
			DpucMGP (pmol/L)	↓ 27%
17 HD patients, 59 y, [68], non- placebo controlled	135 μg/day MK-7	6 weeks	DpcMGP (pmol/L)	-
	45 μg/day MK-7			↓ 17.9%
	135 μg/day MK-7			↓ 36.7%
53 HD patients, 64.6 y, [66], randomized, non- placebo controlled	360 μg/day MK-7	6 weeks	DpucMGP (pmol/L)	↓ 61.1%
	360 μg, thrice weekly MK-7			↓ 17%
	720 μg, thrice weekly MK-7			↓ 33%
200 HD patients, 70.8 y, [62], randomized, prospective, single-blind	1080 μg, thrice weekly MK-7	8 weeks	DpucMGP (pmol/L)	↓ 46%
7 HD patients, 75 y, [142], observational	Stop VKA	5 days	DpucMGP (pmol/L)	↓ 40%
	90 μg/day MK-7		DpucMGP (pmol/L)	↓ 19%
	+10 μg/day		MGP (pg/mL)	↑
	vitamin D			
	10 μg/day vitamin D		DpucMGP (pmol/L)	↑
42 CKD patients, stages 3–5, 58y, [143], Randomized, non-placebo controlled		9 months	MGP (pg/mL)	↓
	90 μg/day MK-7			↓ 10.7%
	+10 μg/day			
	vitamin D			
42 CKD patients, stages 4–5, 58 y, [72], randomized, double-blind	10 μg/day vitamin D	270 days	DpucMGP (pmol/L)	↑
50 HD patients, 64.6 y, [94], pre–post intervention	360 μg/day MK-7	4 weeks	DpucMGP (pmol/L)	↓ 86%
**High CVD risk and HF patients**				
			DpMGP (nmol/L)	
			PMGP (nmol/L)	
			UcMGP (pmol/L)	
1 Keutel syndrome patient, 21 y, [108], case-report, observational	10 mg/day K1	3 months	CMGP (pmol/L)	-
				↓ 45%
	2 mg/day K1			
72 AVC patients, 69.1 y, [144], single-center, open-label	placebo	12 months	DpucMGP (pmol/L)	↑

MGP: matrix Gla protein; ucMGP: uncarboxylated MGP; dpucMGP: dephosphorylated, uncarboxylated MGP; dpcMGP: dephosphorylated, carboxylated MGP; dpMGP: dephosphorylated MGP; pMGP: phosphorylated MGP; cMGP: carboxylated MGP; MK-7: menaquinone-7; VKA: vitamin K antagonist; CKD: chronic kidney disease; HD: hemodialysis; CVD: cardiovascular disease; HF: heart failure; AVC: aortic valve calcification. The percentages reflect change of circulating MGP forms from baseline. - = no statistically significant difference, ↑ = statistically significant positive association, ↓ = statistically significant negative association.
